# Recent advances in bilirubin photoisomerization and photodegradation: in vitro and in vivo evidence for jaundice treatment

**DOI:** 10.1007/s10103-026-04960-y

**Published:** 2026-07-22

**Authors:** Larissa N. Castro, Rafael Ravagnani, Iara R. Tosta, Osmar V. da Silva, Lucas D. Dias

**Affiliations:** Universidade Evangélica de Goiás, Anápolis, Brazil

**Keywords:** Photoisomerization, Phototherapy, Neonatal hyperbilirubinemia, Light-mediated therapy

## Abstract

Neonatal jaundice, characterized by unconjugated hyperbilirubinemia, remains one of the most prevalent conditions affecting both term and preterm neonates. Elevated bilirubin levels may progress to acute bilirubin encephalopathy or kernicterus, resulting in irreversible neurological damage and increased mortality. Phototherapy is the standard treatment for neonatal hyperbilirubinemia, promoting bilirubin elimination through photoisomerization and photodegradation pathways that generate more water-soluble products, bypassing hepatic metabolism. Over the past decades, significant advances have been made in light-based technologies aimed at improving the efficiency of bilirubin photoconversion. In particular, wavelength optimization, high-intensity light-emitting diodes (LEDs) systems, and emerging photonic and nanomaterial-based approaches have been explored to enhance therapeutic outcomes. In this review, a structured literature search was conducted using the Web of Science database, covering the past 20 years, to analyze recent developments in bilirubin photoisomerization and photodegradation (in vitro and in vivo studies). The results demonstrate that photodegradation efficiency is strongly dependent on wavelength, with optimal performance observed in the blue-green region (490–500 nm), where reduced bilirubin half-life and increased lumirubin formation are reported. Furthermore, high-intensity LED systems generally provide higher irradiance and more favorable degradation kinetics than conventional light sources, although direct comparison among studies remains limited by differences in irradiation geometry, exposure time, bilirubin concentration, and experimental models. Emerging strategies, including optoacoustic monitoring, fluorescence-based theragnostic systems, and nanomaterial-assisted photocatalysis (e.g., ZnO nanoparticles, Au nanocomposites, and functionalized Fe₃O₄ systems), have demonstrated enhanced bilirubin degradation and offer new perspectives for both therapeutic optimization and real-time monitoring. However, most of these approaches remain at the proof-of-concept or preclinical stage, and their clinical translation requires validation of biocompatibility, selectivity, reproducibility, scalability, and neonatal safety. Optical and point-of-care sensing technologies may enable individualized adjustment of phototherapy, but their performance must be validated under clinically relevant conditions and across different gestational ages, skin characteristics, and bilirubin concentrations. Additionally, bilirubin degradation is not solely a detoxification process, as photooxidation products may exhibit biological activity with potential clinical implications. Future studies should therefore focus on optimizing irradiation parameters (wavelength, irradiance, and exposure time), elucidating photochemical pathways, and characterizing degradation products, while correlating these findings with clinical outcomes. Standardized experimental and clinical protocols will also be essential to compare technologies and determine whether improved photochemical performance translates into faster bilirubin reduction, shorter treatment duration, and fewer adverse effects. Such integration may support the development of more efficient, safer, and personalized phototherapy strategies in neonatal care.

## Introduction

Neonatal jaundice is one of the most common causes of hospitalization in the first week of life worldwide [1]. About 60% of term newborns and 80% of preterm newborns present jaundice within the first 3 days of life [2]. It is characterized by high levels of unconjugated bilirubin in the blood, called hyperbilirubinemia, caused by several mechanisms [2]. Bilirubin is mainly generated by the destruction of red blood cells, in which increased formation of this yellow substance and the inability of the liver to process it result in its accumulation, consequently in its deposition in tissues, causing the yellowish discoloration of the skin, mucous membranes, and sclera, known as jaundice [3]. Due to the immature hepatic metabolism of neonates, the capacity to process and excrete bilirubin is reduced [4]; therefore, high levels may present significantly high risks, as they cross the blood-brain barrier and deposit in certain regions of the brain, progressing to acute bilirubin encephalopathy (ABE) or kernicterus, causing sequelae with mortality risk in newborns [5–7]. Kernicterus is the chronic and permanent form of bilirubin encephalopathy, whose characteristic is irreversible neurological damage such as cerebral palsy, auditory neuropathy, and intellectual disability, caused by the deposition of bilirubin in brain nuclei [8], and may progress to death.

Bilirubin is formed as a degradation product of heme, intensified in the neonatal period due to accelerated physiological hemolysis, in which newborns present a red blood cell turnover rate 2 to 3 times higher than adults because of the reduced lifespan of erythrocytes [9]. Furthermore, neonates present limited hepatic capacity due to liver immaturity, in which deficiency of the conjugating enzyme (UGT1A1), responsible for converting indirect bilirubin (lipid-soluble) into direct bilirubin (water-soluble and excretable), has its activity reduced in the first days of life [10, 11]. Because indirect bilirubin is lipid-soluble, it is easily reabsorbed by the intestinal mucosa and returns to the blood, forcing the liver to process the same bilirubin repeatedly. Newborns present increased enterohepatic circulation, in which one of the factors responsible is the enzyme β-glucuronidase present in breast milk, which deconjugates direct bilirubin back into indirect bilirubin, which is reabsorbed and returns to the bloodstream [12]. Another factor is the absence of adequate bacterial flora; bacteria convert bilirubin into non-absorbable substances (stercobilinogen) to be excreted in feces, but as they are not yet established in the newborn’s organism, bilirubin does not have an efficient pathway for excretion, favoring enteral reabsorption [13]. In addition, there are conditions that may aggravate hyperbilirubinemia [11], such as prematurity, blood incompatibilities, G6PD deficiency, etc. Prematurity is a significant risk factor, considering that the liver is even more immature than that of a term infant, further aggravating the situations previously described [14, 15]. ABO/Rh blood incompatibility may cause extravascular hemolysis in the fetus or newborn, mediated by maternal antibodies that cross the placenta [16]. Glucose-6-phosphate dehydrogenase (G6PD) deficiency is the most common genetic defect, affecting millions of people worldwide [17]. This enzyme is essential for erythrocyte stability; its reduced production allows red blood cells to become more susceptible to oxidative stress, potentially resulting in acute hemolysis, anemia, and jaundice [18].

**Fig. 1:**
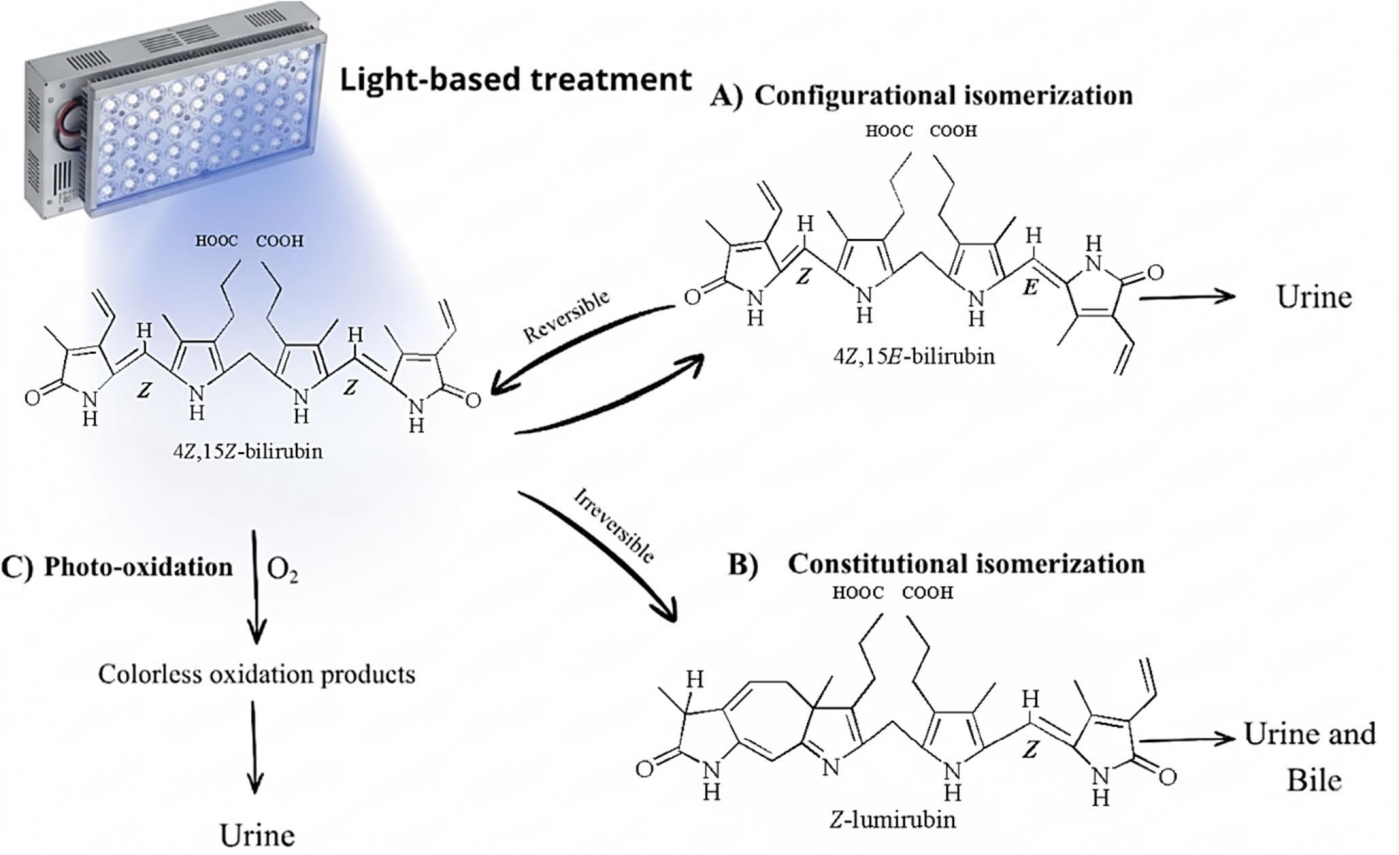
Photochemical pathways of bilirubin during phototherapy. Schematic representation of the molecular transformations of native 4*Z*,15*Z*-bilirubin induced by blue light irradiation. The process involves three primary mechanisms: (**A**) Reversible configurational isomerization to 4*Z*,15*E*-bilirubin; (**B**) Constitutional isomerization to *Z*-lumirubin, both resulting in more polar isomers that facilitate excretion via urine and/or bile; and (**C**) Photooxidation, a slower reaction involving oxidative cleavage of the bilirubin molecule. This pathway yields acyclic, highly water-soluble polar products that are predominantly excreted in the urine. Adapted from [24]

Phototherapy is the primary treatment for hyperbilirubinemia. It induces photochemical reactions that convert bilirubin into water-soluble isomers that can be excreted without additional hepatic conjugation [19]. Photoisomerization is the fastest and primary pathway for bilirubin elimination and involves three main mechanisms: configurational (*Z*–*E*) photoisomerization, structural (constitutional) isomerization leading to lumirubin formation, and photooxidation. Configurational (*Z*–*E*) photoisomerization involves the conversion of the natural *Z*,* Z* isomer into *E*,*Z* or *E*,*E* forms, which are more water soluble but reversible upon cessation of irradiation, potentially reverting to the *Z*,*Z* configuration [20, 21]. Structural (constitutional) isomerization leads to the formation of lumirubin, a highly polar compound that is rapidly excreted in urine. This pathway is considered a major contributor to phototherapy efficacy because lumirubin is rapidly eliminated and is not expected to undergo appreciable reconversion under physiological conditions, although reverse photoisomerization has been demonstrated under specific experimental conditions [22–25]. Photooxidation is a slower reaction in which bilirubin oxidation results in oxidative cleavage, yielding polar and acyclic molecules that are highly water soluble and predominantly excreted in urine [26, 27]. This process is illustrated in Fig. 1.

The discovery of phototherapy dates to the 1950s, when nurses at Rochford General Hospital observed visible reductions in jaundice among infants exposed to sunlight. In 1958, Cremer and R.W. Perryman published studies demonstrating that blue light emitted by 40 W “light blue” fluorescent lamps effectively reduced serum bilirubin levels in newborns [28]. Since then, technological advancements have been increasingly focused on improving safety and therapeutic efficiency. Blue, fluorescent lamps were introduced to provide adequate irradiance within the optimal wavelength range (≈ 460–490 nm), although these devices emitted heat and had a relatively short lifespan. Halogen lamps offered high-intensity illumination but produced excessive heat, limiting their clinical use. Between the 1990s and 2000s, light-emitting diodes (LEDs) revolutionized phototherapy by delivering light within the most effective band for photodegradation while maintaining lower heat emission, longer lifespan, and stable irradiance, becoming the current standard of care [29–31]. Additional innovations, such as biliblankets using optical fiber in pads and wraps, deliver blue light directly to the neonatal skin, enabling effective treatment with greater comfort and mobility [32].

Despite significant advancements, substantial variability persists in the effectiveness of different phototherapy devices and light sources. Ongoing debates concern the optimal wavelength, irradiance, light-field homogeneity, and exposure duration required for effective reduction of serum bilirubin levels. In this context, an updated review of the methods, mechanisms of action, advantages, limitations, and safety considerations of phototherapy is warranted. The relevance of such a review is broad, encompassing neonatology, biomedical engineering, responsible for device development and optimization and public health, given the implications for morbidity, mortality, and healthcare costs.

## Methodology

A structured literature search was performed in the Web of Science database to identify studies published over the last 20 years addressing bilirubin photodegradation. The search strategy used the terms “photodegradation” AND “bilirubin”, applied to titles, abstracts, and keywords. The search was restricted to articles published in English and subjected to subsequent screening for relevance. Studies were considered eligible when they: (i) investigated bilirubin photodegradation and/or photoisomerization; (ii) reported mechanistic, kinetic, spectroscopic, or experimental aspects related to light-induced bilirubin transformation; (iii) included in vitro and/or in vivo approaches; and (iv) provided sufficient methodological detail regarding irradiation conditions, such as wavelength, light source, irradiance, exposure time, or reaction medium. The following exclusion criteria were applied: (i) studies not directly focused on bilirubin photodegradation or photoisomerization; (ii) articles addressing neonatal jaundice or phototherapy without experimental or mechanistic discussion of bilirubin photochemistry; (iii) conference abstracts, editorials, letters, notes, patents, and book chapters; (iv) duplicate records; and (v) studies with insufficient experimental information for qualitative comparison. After title and abstract screening, potentially relevant articles were assessed in full text. Studies meeting the eligibility criteria were then included in the qualitative analysis, with emphasis on the mechanisms, kinetics, irradiation parameters, and experimental conditions governing bilirubin photodegradation.

## Results and discussion

Table 1 summarizes the in vitro studies, emphasizing the main experimental variables associated with bilirubin phototransformation, including bilirubin concentration, irradiation wavelength, exposure time, reaction medium, and degradation efficiency. To facilitate comparison across studies, the results and discussion section was organized into thematic subsections addressing wavelength and irradiance, light-source effects, photochemical mechanisms, optical monitoring, integrated sensing-therapy platforms, nanomaterial-assisted photodegradation, and in vivo validation.

**Table 1:** Main experimental parameters and degradation efficiencies reported for in vitro bilirubin photodegradation studies

Entry	Bilirubin concentration (µM)	Wavelength (nm)	Duration time (h)	Solvent	Degradation (%)	Ref
1	342	468	0.5	Human serum albumin	28	[33]
2	427	390–530	2	Human serum albumin	50	[34]
3	342	465–470	5	Human albumin, NaOH and Na_2_HPO_4_	37–51	[35]
4	428	390 and 530	0.2–1.0	4% HSA	50	[36]
5	23	620–690	0.2	Tris-buffered aqueous solution	50	[37]
6	NR	476	24	Cerebrospinal fluid samples	50	[38]
7	342	460	4	Chloroform solution / Milli–Q water / Physiological saline	50	[39]
8	13	454	0.2	Bilirubin–HSA solution	100	[40]
9	17–855	455 and 530	0.03	DMSO	13	[41]
10	373	467	2.5	Dulbecco’s Phosphate-Buffered Saline (DPBS)	NR	[42]
11	2–40	450	0.8	DI water	37	[43]
12	NR	490–570	2.3	Water	75	[44]
13	342	450	0.3	Milli–Q water	30	[45]

*NR* not reported

### Wavelength, irradiance, and light-source effects

**Fig. 2:**
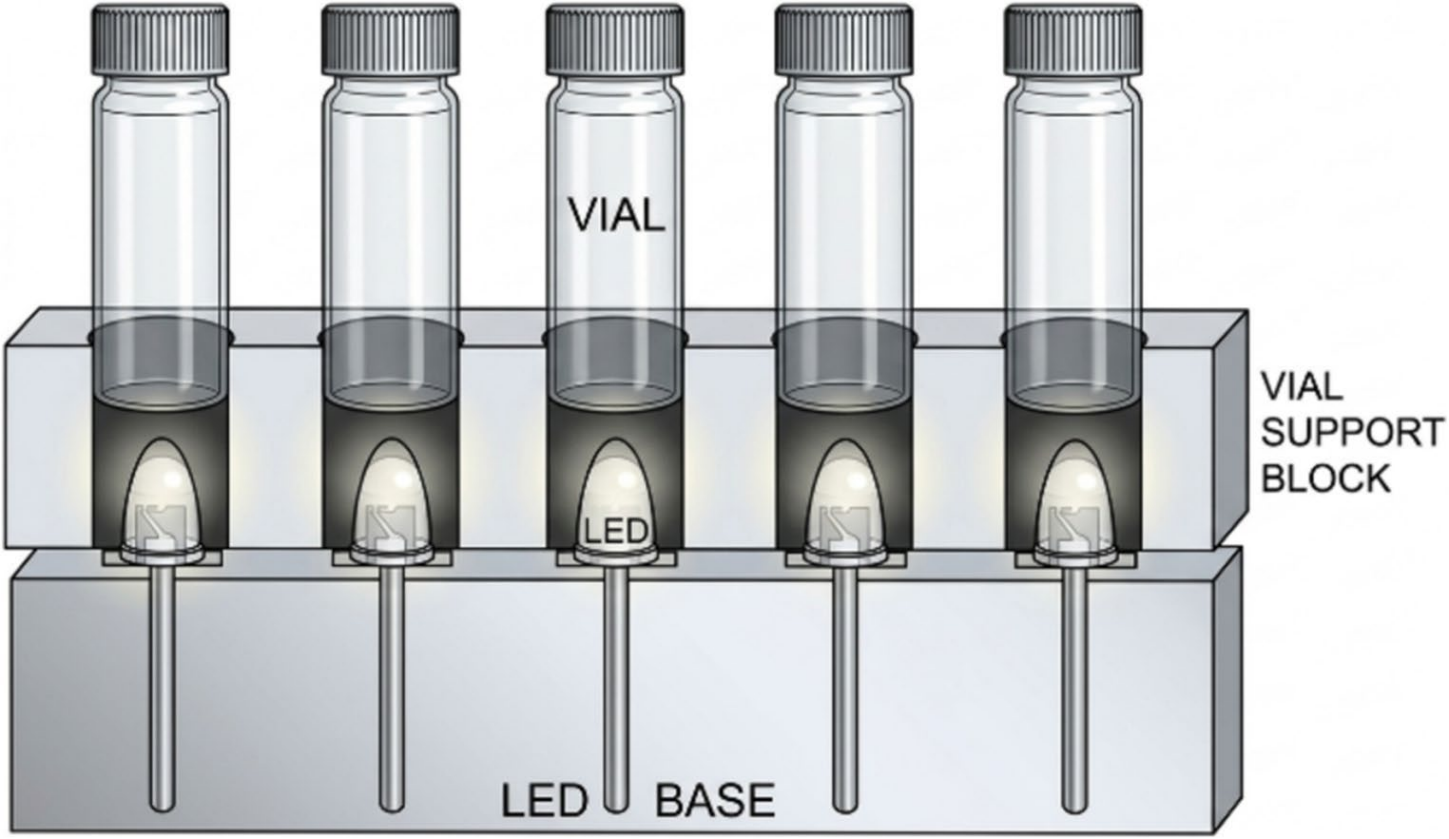
Schematic representation of the experimental LED irradiation device comprising five LEDs mounted on a common base and aligned beneath individual sample vials. A support block maintained the vials in a fixed vertical position, with their bottoms placed directly above the LEDs to ensure localized bottom-up irradiation of the bilirubin solutions. Adapted from [33].

In 1998, Vreman and colleagues [33] investigated high-intensity light-emitting diodes (LEDs) as a novel light source for phototherapy in the treatment of neonates with hyperbilirubinemia and compared their efficacy with conventional fluorescent light sources used at the time (Fig. 2; Table 1, entry 1). They tested individual LEDs of different colors and subsequently constructed a prototype phototherapy device composed of 300 blue LEDs, measuring in vitro bilirubin photodegradation in human serum albumin under light irradiation. Among the different colors tested, blue light showed the highest efficacy in promoting bilirubin degradation, followed by blue-green and white light, whereas green light was the least effective. The prototype device, consisting of three focused arrays of 100 blue LEDs, generated an irradiance exceeding 200 µW·cm⁻²·nm⁻¹, higher than that produced by then-standard fluorescent phototherapy units, and supported a higher rate of bilirubin photodegradation. According to the authors, LED-emitted light represented a promising alternative to the fluorescent phototherapy sources. However, because the study was conducted in vitro, these findings did not directly establish clinical efficacy or neonatal safety.

In 2019, Vreman and colleagues [34] systematically investigated the effect of light wavelength on in vitro bilirubin photodegradation and photoisomer production using a controlled LED-based irradiation system with narrow wavelength ranges (Table 1, entry 2). Bilirubin solutions bound to human serum albumin were exposed to normalized photon flux across wavelengths ranging from 390 to 530 nm, and bilirubin half-life times as well as photoisomer profiles were quantitatively determined. Normalization of photon flux enabled a direct comparison of wavelength effects, minimizing irradiance-related bias. The results demonstrated a progressive increase in photodegradation efficiency with longer wavelengths, with the shortest bilirubin half-life and highest lumirubin production occurring between 490 and 500 nm. Additionally, bilirubin photodegradation at 37 °C showed a linear relationship with wavelength, with half-life decreasing from 63 min at 390 nm to 17 min at 500 nm. At 460 ± 10 nm, a lower photodegradation rate and higher half-life (31 min) were observed compared to 500 nm (17 min). Spectroscopic and chromatographic analyses confirmed that lumirubin formation was the dominant pathway of bilirubin photodegradation and closely correlated with degradation kinetics. These findings challenge the assumption that the conventional 460 nm range is necessarily optimal when photon delivery is controlled. However, the albumin-based solution does not reproduce tissue absorption and scattering, limiting direct extrapolation to neonatal phototherapy. Therefore, carefully designed in vivo studies to evaluate whether such wavelength optimization could improve clinical phototherapy.

**Fig. 3:**
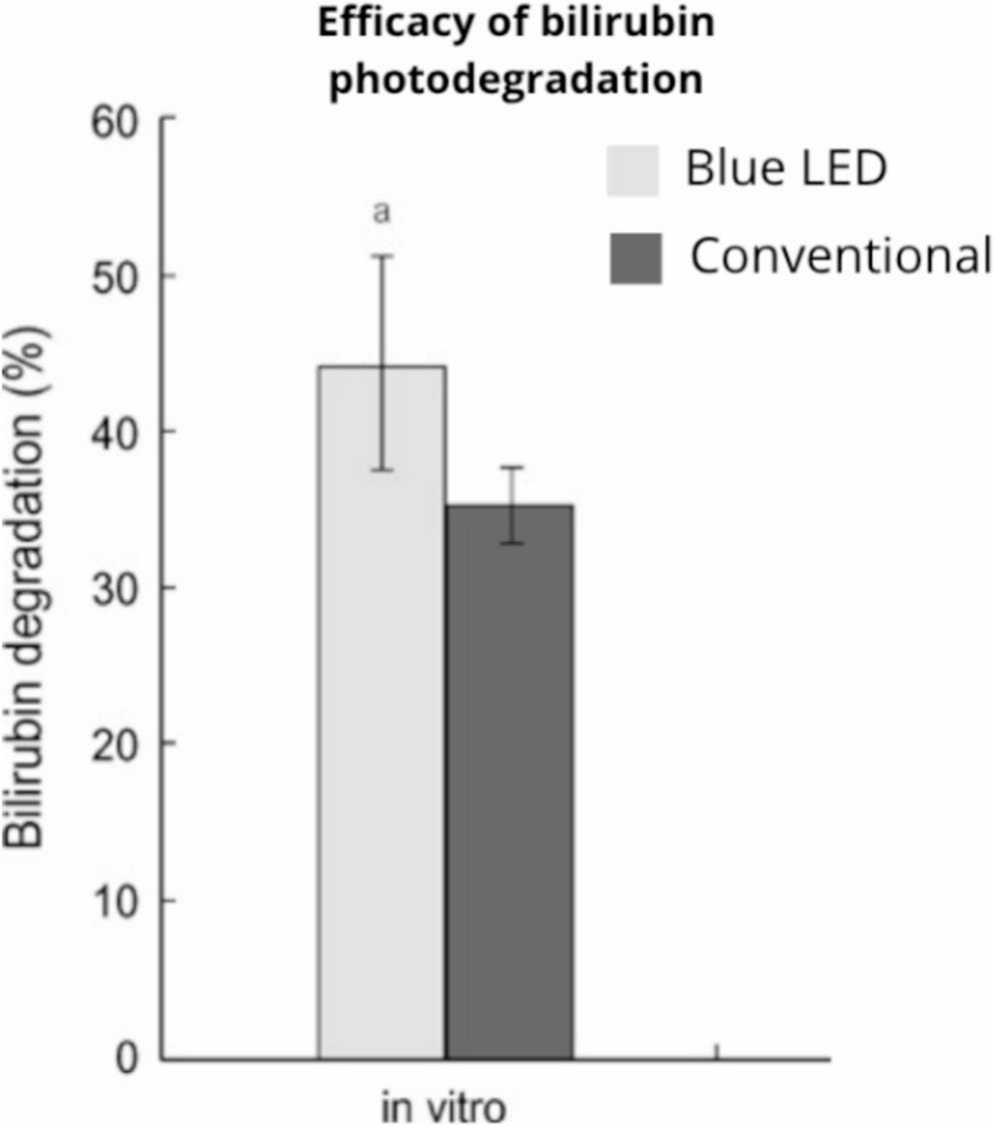
In vitro bilirubin photodegradation achieved using a high-intensity blue LED system compared with a conventional quartz–halogen phototherapy device. Bars represent the mean percentage of bilirubin degradation, and error bars indicate the standard deviation. The blue LED system produced significantly greater bilirubin degradation than the conventional device. **ᵃ** *p* < 0.05 versus the conventional system. Adapted from [35].

In 2005, Chang et al. [35]. evaluated in vitro performance the efficacy of a high-intensity blue gallium nitride (GaN) LED phototherapy prototype for bilirubin photodegradation in comparison with a conventional quartz–halogen device (Fig. 3; Table 1, entry 3). The in vitro experiments were conducted using microhematocrit tubes containing bilirubin solutions, a simplified model that does not reproduce the optical and biological complexity of neonatal tissues. The LED system consisted of two focused arrays, each comprising 500 blue LEDs emitting within a narrow spectral range (465–470 nm), closely matching the absorption peak of bilirubin. This configuration generated higher irradiance levels while producing less heat than the conventional system, resulting in enhanced bilirubin photodegradation. However, because wavelength distribution and irradiance differed simultaneously between the devices, the contribution of each parameter could not be independently determined. The improved performance was primarily attributed to the combination of wavelength specificity and increased irradiance.

These studies indicate that bilirubin phototransformation depends on the interaction between wavelength, irradiance, spectral bandwidth, and experimental medium. Although wavelengths near 490–500 nm produced faster photodegradation under normalized photon flux, systems operating near 460–470 nm also showed high performance when higher irradiance was delivered. Therefore, the reported optimal wavelength cannot be interpreted independently of photon delivery and experimental design.

### Photoisomerization, photooxidation, and medium effects

In 2000, Kranc and colleagues [36] investigated the oxidative degradation of bilirubin under non-enzymatic conditions and demonstrated that this process leads to the formation of biologically active degradation products (Table 1; entry 4). By exposing bilirubin to oxidative agents in vitro, Kranc et al. showed that bilirubin breakdown generates compounds capable of inducing vasoactive effects, including increased oxygen consumption and contractile responses in vascular smooth muscle. Chemical characterization revealed a mixture of bilirubin oxidation products, some of which are related to known biliverdin degradation pathways. According to the authors, these findings indicate that bilirubin degradation is not merely a detoxification process but may also contribute to vascular dysfunction under pathological conditions associated with oxidative stress and bilirubin accumulation, such as subarachnoid hemorrhage. However, the relevance of these chemically generated oxidation products to neonatal phototherapy remains to be established.

In 1996, Jirsa and colleagues [37] investigated the photodegradation of bilirubin ditaurate (BDT) in aqueous solutions in the presence of different photosensitizers (Table 1, entry 5), with emphasis on the role of singlet oxygen and the potential relevance of this system to photodynamic therapy (PDT). The results showed that the rate constant of BDT photodegradation increases with dissolved oxygen concentration and in D₂O-containing media, indicating that singlet oxygen–mediated oxidation is the predominant mechanism. Among the photosensitizers tested, thiazine dyes, particularly methylene blue and azur II, exhibited the highest photodegradation rates, attributed to their intense absorption bands above 600 nm. These findings indicate that BDT is a suitable substrate for singlet oxygen detection and highlight the potential of thiazine dyes for PDT applications using conventional light sources. However, because the study was performed in a simplified aqueous model, its direct therapeutic relevance remains limited.

In 2010, Foroughi and colleagues [38] investigated the influence of light exposure and storage time on bilirubin degradation in cerebrospinal fluid (CSF) samples analyzed by spectrophotometry for the diagnosis of subarachnoid hemorrhage (SAH) (Table 1, entry 6). CSF samples from patients with CT-positive SAH were divided into two cohorts and stored either under normal room light or protected from light, with serial spectrophotometric measurements performed up to 48 h after collection. Net bilirubin absorbance was determined at 476 nm, allowing quantification of degradation kinetics under both conditions. The results demonstrated that bilirubin degradation occurred in most samples and was significantly faster in light-exposed specimens compared with those stored in the dark, although the marked variability between samples limits the use of a single correction factor for storage-related degradation. Importantly, a clinically relevant reduction of bilirubin absorbance below diagnostic thresholds occurred more frequently in samples exposed to light, directly increasing the risk of false-negative interpretation. According to the authors, bilirubin in CSF is susceptible to unpredictable photodegradation, underscoring the need for rapid analysis, immediate centrifugation, protection from light, and careful consideration of storage time when interpreting CSF spectrophotometry results for SAH diagnosis.

In 2015, Cardoso and colleagues [39] evaluated the effects of blue light (460 nm) on bilirubin, emphasizing the roles of photoisomerization and photooxidation during neonatal phototherapy (Table 1, entry 7). UV–Vis and FTIR spectroscopy, combined with quantum molecular modeling, were used to analyze bilirubin in chloroform, ultrapure water, and physiological saline. The results show that, in addition to photoisomerization, bilirubin undergoes significant photooxidation within the first hour of irradiation, evidenced by a progressive decrease in absorbance and structural modifications detected by FTIR. Nevertheless, these spectroscopic changes do not independently quantify the relative contribution of each pathway. Bilirubin degradation was faster in aqueous media, likely due to higher oxygen availability, and was partially delayed in saline solutions, demonstrating that solvent composition strongly affects the observed degradation kinetics and may explain differences among studies. Theoretical calculations indicate that the (4*Z*,15*E*) isomer exhibits higher polarity and aqueous solubility, explaining its more efficient excretion during phototherapy. These findings demonstrate that bilirubin elimination involves the combined contribution of photoisomerization and photooxidation.

These findings show that the relative contribution of photoisomerization and photooxidation depends strongly on oxygen availability, solvent composition, protein binding, and irradiation conditions. Photoisomerization appears to predominate under conventional phototherapy conditions, whereas oxidative pathways become more relevant in oxygen-rich media, photosensitized systems, and prolonged irradiation. Direct comparison among studies remains limited by differences in analytical methods and biological matrices.

### Photodegradation kinetics as an analytical tool for bilirubin sensing

Controlled bilirubin photodegradation can be exploited as an analytical tool because irradiation-induced changes in optical absorption, fluorescence, and optoacoustic signals depend on the initial bilirubin concentration and evolve predictably over time. Recent approaches have used these kinetic responses either independently or in combination with multiwavelength photometry, particularly blue and green optical channels, to provide complementary spectral and temporal information for bilirubin quantification. This multimodal strategy has enabled the development of optoacoustic systems, fluorescence-based theragnostic approaches, and miniaturized system-on-chip platforms capable of analyzing very small blood volumes and supporting continuous or point-of-care monitoring. By integrating sensing with controlled irradiation, these platforms may improve measurement reliability and facilitate real-time assessment during phototherapy [41, 46, 47].

**Fig. 4:**
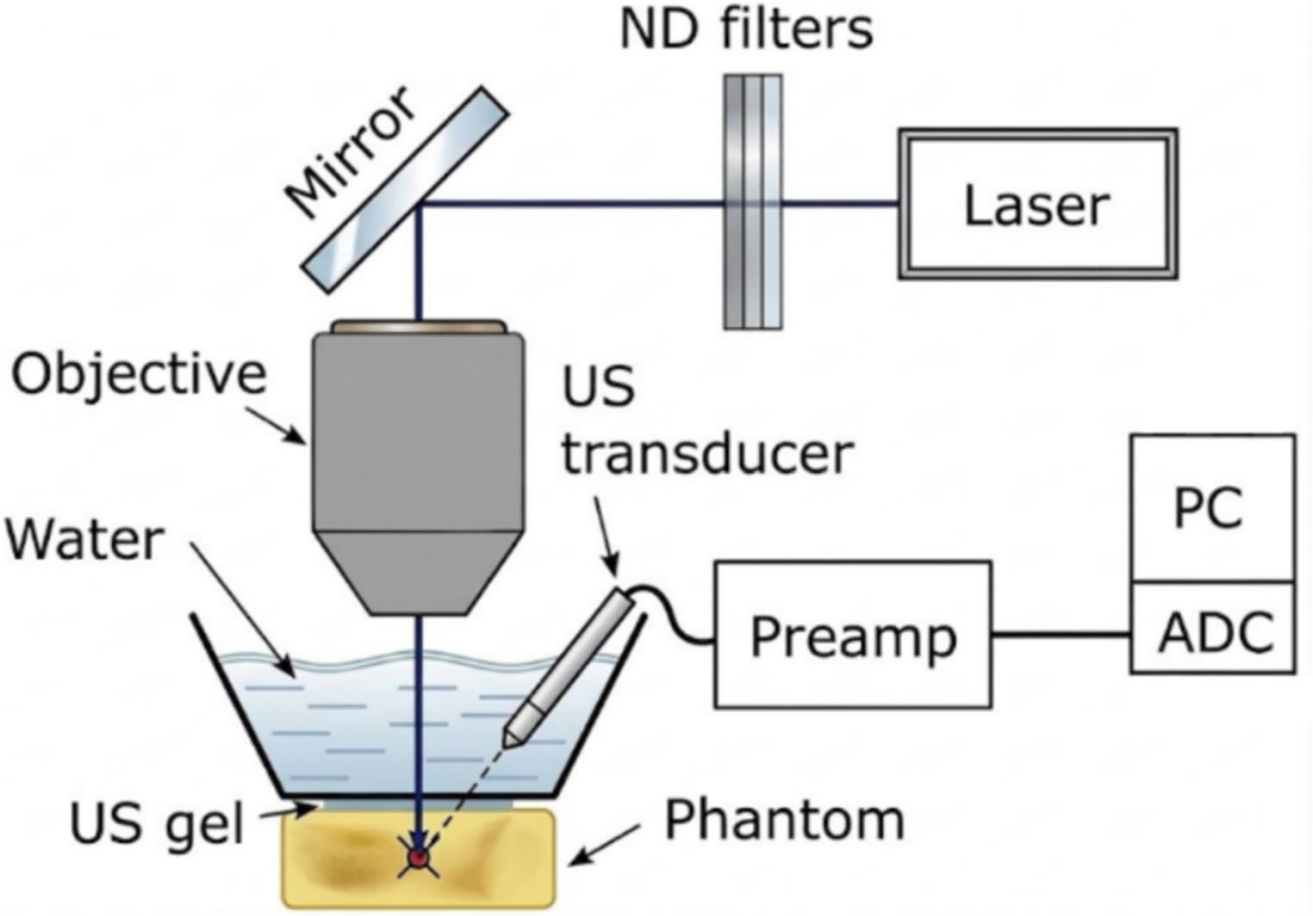
Schematic representation of the optoacoustic system used to characterize bilirubin-containing skin phantoms under 470 nm laser irradiation. The laser beam was attenuated using neutral-density (ND) filters, redirected by a mirror, and focused onto the phantom through an objective lens. The generated optoacoustic signals were detected by an ultrasound (US) transducer coupled to the sample through water and ultrasound gel, amplified by a preamplifier, digitized using an analog-to-digital converter (ADC), and processed on a computer. Adapted from [40].

In this regard, in 2023, Perkov and colleagues [40] developed gelatin-based phantoms to study bilirubin photodegradation under blue light and built a custom optoacoustic device to monitor this process (Fig. 4; Table 1, entry 8). To demonstrate detection at depth, they fabricated a multilayer gelatin phantom with a set of layers with distinct bilirubin concentrations and evaluated optoacoustic responses after blue light laser irradiation (at 470 nm). The system showed an exponential decrease in optical extinction and optoacoustic signal with irradiation time and enabled construction of a calibration curve for bilirubin quantification. According to the authors, blue light effectively promotes bilirubin photodegradation, and optoacoustic spectroscopy real-time, depth-resolved monitoring of bilirubin photodegradation. However, the study was conducted using simplified gelatin phantoms that do not fully reproduce the optical scattering, vascularization, pigmentation, and biological complexity of neonatal tissues. Therefore, although this approach represents a promising proof of concept, its clinical applicability requires in vivo validation, assessment of measurement accuracy across different tissue depths and skin characteristics, and comparison with established transcutaneous and serum bilirubin measurements. According to the authors, optoacoustic spectroscopy could potentially support bilirubin monitoring during phototherapy, particularly in neonatal jaundice and subcutaneous hematomas.

**Fig. 5:**
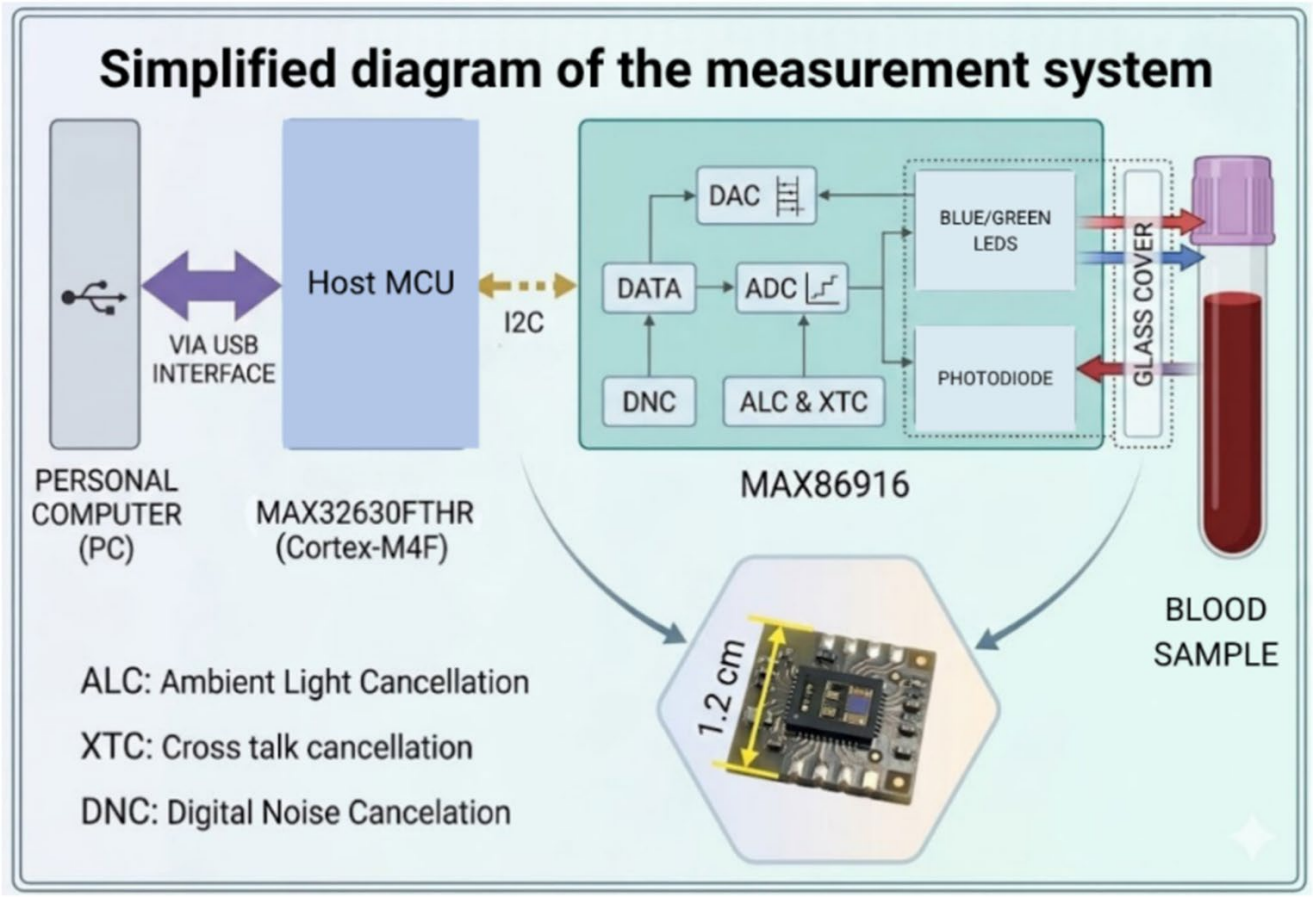
Schematic of the miniaturized bi-modal bilirubin monitoring system based on the MAX86916 sensor, integrating blue/green LEDs, a photodiode, signal processing, and data transfer to a host microcontroller and computer. Adapted from [41].

In 2023, Ndabakuranye and colleagues [41] developed a miniaturized bi-modal system-on-chip (SoC) platform for bilirubin monitoring by combining dual-wavelength photometric measurements with photodegradation kinetics (Fig. 5; Table 1, entry 9). The system integrated blue (455 nm) and green (530 nm) LEDs, a photodiode, and signal-processing electronics within a compact MAX86916 chip (3.5 × 7.0 mm), enabling measurements using less than 10 µL of whole blood. This dual approach may improve analytical robustness by combining static optical information with a kinetic response. Bilirubin concentration was extracted by monitoring both the ratio of optical densities at two wavelengths and the temporal evolution of optical signals during controlled photodegradation. Experiments performed on porcine blood samples spanning pathophysiological bilirubin levels (1–50 mg.dL^− 1^) demonstrated strong correlations between bilirubin concentration and both photometric and degradation-derived parameters (R² > 0.95), with average accuracies of approximately ± 10–13%. The use of porcine blood and the absence of comparison with a reference clinical method limit conclusions regarding performance in human neonatal samples. According to the authors, the integration of photometric and photodegradation approaches within a single SoC platform represents a promising proof of concept for low-cost, portable point-of-care bilirubin monitoring, particularly for applications outside conventional laboratories and hospital settings.

In 2023, Perkov and Gorin [42] developed a noninvasive and continuous method for monitoring bilirubin photodegradation during phototherapy, with a focus on its application in neonatal jaundice (Table 1, entry 10). The results showed that the photochemical degradation of bilirubin leads to a reduction in optical absorption and characteristic fluorescence behavior over time, which strongly depends on the initial bilirubin concentration. At low concentrations, fluorescence reached a maximum rapidly, within approximately 30 min, whereas at high, the maximum was not reached even after 2.5 h of irradiation. This behavior was attributed to different fluorescence self-quenching mechanisms, mainly related to reabsorption of emitted light by bilirubin molecules bound to the same or to neighboring albumin molecules. The study demonstrated a linear relationship between bilirubin concentration and the time required to reach maximum fluorescence intensity, enabling the construction of calibration curves. The authors conclude that fluorescence-based monitoring can be integrated with the therapeutic light source itself, forming a theragnostic approach that may improve the accuracy of current noninvasive methods when combined with other optical techniques.

Together, these technologies demonstrate a transition from isolated bilirubin measurements toward continuous or point-of-care monitoring during phototherapy. Nevertheless, most systems remain at the proof-of-concept stage and require validation in neonatal samples, comparison with reference clinical methods, and evaluation of tissue- and matrix-related interferences.

### Nanomaterial-assisted photodegradation

**Fig. 6:**
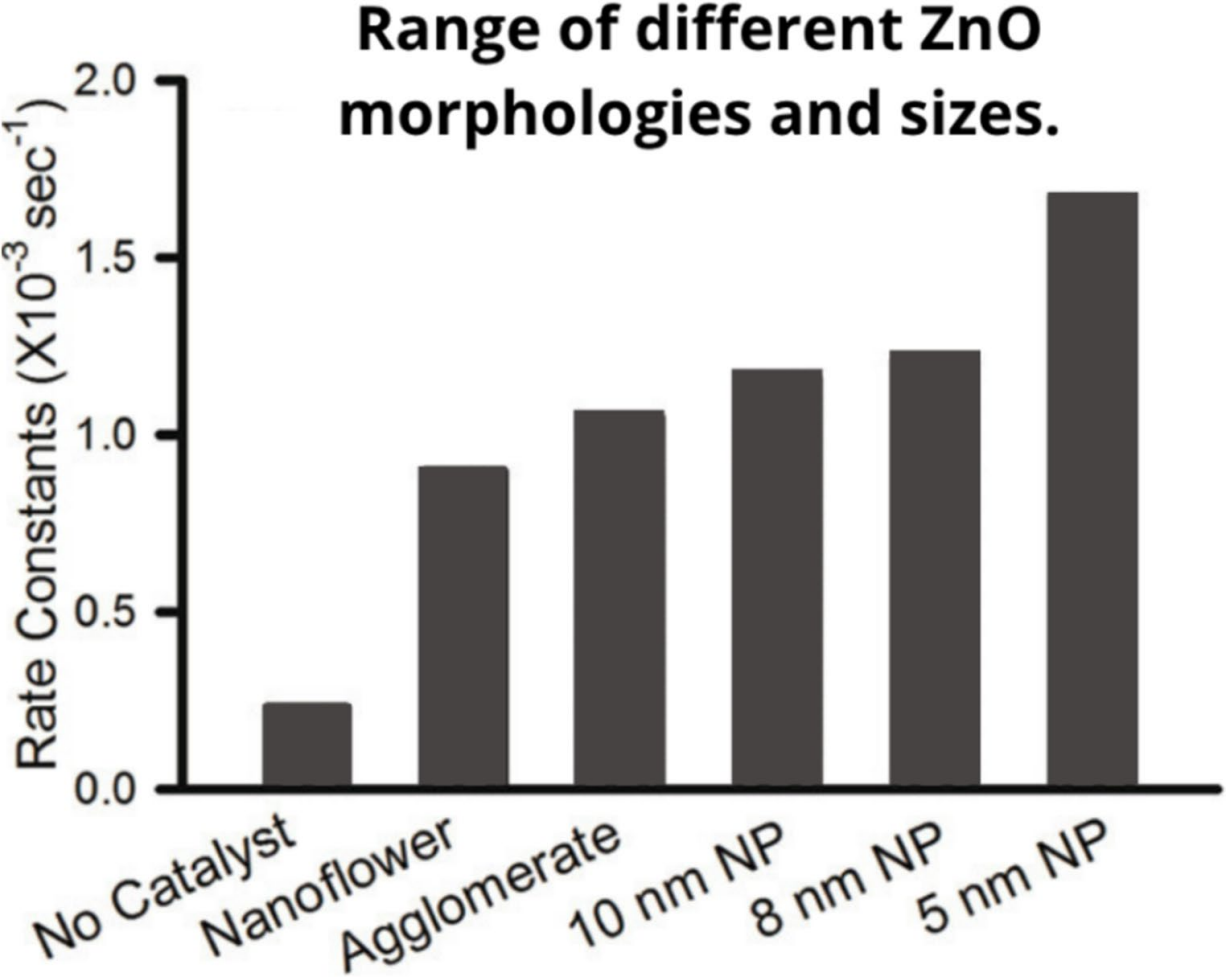
Catalytic rate constants for bilirubin photodegradation using ZnO catalysts with different morphologies and particle sizes. The 5 nm ZnO nanoparticles exhibited the highest catalytic activity. Adapted from [43].

In 2012, Sarkar and colleagues [43] investigated the photocatalytic degradation of bilirubin using zinc oxide (ZnO) nanoparticles with different sizes and morphologies (Fig. 6; Table 1, entry 11). The authors demonstrate that ZnO nanoparticles with diameters of approximately 5 nm, rich in defect states associated with oxygen vacancies, exhibit the highest catalytic efficiency, achieving about 37% degradation in 3000 s. The proposed mechanism is based on nonradiative Förster resonance energy transfer (FRET) between defect states of ZnO and bilirubin adsorbed on the nanoparticle surface, promoting its degradation under UV irradiation. The degradation kinetics follow a pseudo-first-order model, consistent with the Langmuir–Hinshelwood mechanism. In addition, the nanoparticles proved to be reusable and effective in flow systems, including the degradation of bilirubin bound to human serum albumin, without significant alterations to the protein structure. Although these findings support the photocatalytic potential of ZnO, the dependence on UV irradiation and the absence of biological safety evaluation limit direct therapeutic translation to neonatal phototherapy.

**Fig. 7:**
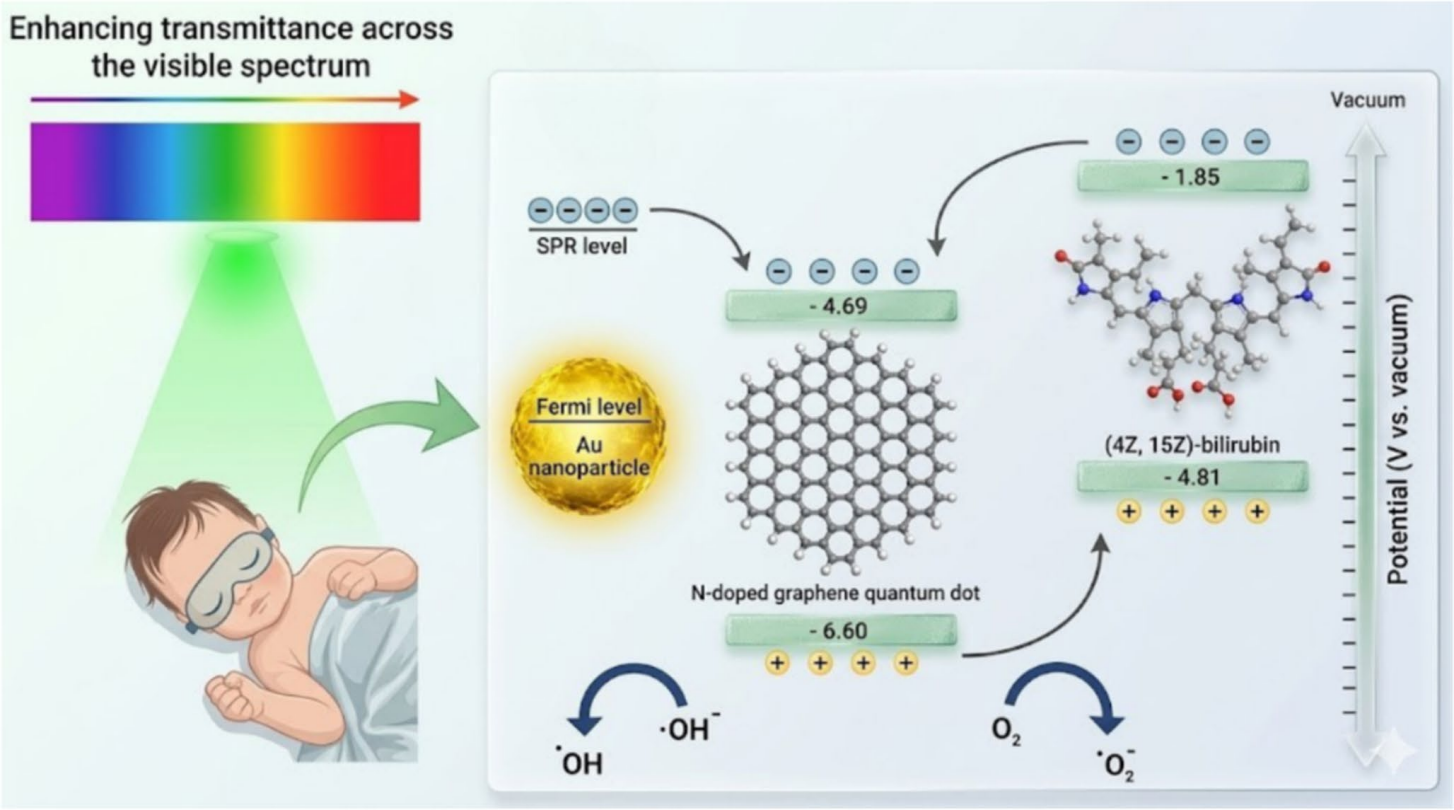
Proposed photocatalytic mechanism of the NGQDs/Au nanocomposite under visible-light irradiation, involving plasmon-assisted charge transfer, reactive oxygen species generation, and bilirubin degradation. Adapted from [44].

In 2024, Dejpasand and colleagues [44] developed a biocompatible nanocomposite composed of nitrogen-doped graphene quantum dots and gold nanoparticles (NGQDs/Au for the photocatalytic degradation of bilirubin under green light irradiation (Fig. 7; Table 1, entry 12). The material was synthesized via a hydrothermal method followed by a photochemical route and characterized using structural, morphological, and optical techniques, confirming nanocomposite formation and the presence of the surface plasmon resonance effect of Au. The results indicated that the incorporation of gold nanoparticles promotes suppression of electron–hole recombination, thereby enhancing photocatalytic activity. The NGQDs/Au nanocomposite demonstrated high apparent bilirubin removal under green light irradiation, with near-complete removal achieved when dark adsorption and illuminated degradation were considered together. Therefore, the contribution of photocatalysis should be distinguished from adsorption when interpreting the overall efficiency. The proposed mechanism involves charge transfer between bilirubin and NGQDs, as well as the generation of reactive species mediated by plasmonic excitation of gold nanoparticles. Unlike conventional blue-light phototherapy, which predominantly promotes bilirubin photoisomerization, this system relies mainly on plasmon-assisted photooxidation. Consequently, direct claims of superiority are limited by differences in mechanism and experimental conditions. These findings highlight the potential of NGQDs/Au as a green-light photocatalyst, although validation in biological matrices and further safety assessment are required before therapeutic application.

In 2014, Goswami and colleagues [45] developed a top-down surface engineering strategy for magnetite (Fe₃O₄) nanoparticles, using tartrate as a functionalizing ligand, with the aim of inducing new optical and catalytic properties and applying them to bilirubin degradation (Table 1, entry 13). In this process, Fe₃O₄ nanoparticles with an initial diameter of approximately 23 nm, originally dispersed in an organic medium, were transferred to the aqueous phase through ligand exchange and subjected to a *core etching* process, resulting in smaller particles (~ 5 nm). The functionalized nanoparticles (T-Fe₃O₄) exhibited ligand-to-metal charge transfer (LMCT) transitions in the UV region, as well as intense blue luminescence (properties absent in the parent nanoparticles). The authors demonstrated that these nanoparticles display efficient and reusable photocatalytic activity, enabling bilirubin degradation under UV irradiation through mechanisms associated with excited-state energy and electron transfer. This mechanism differs from conventional blue–green phototherapy, which primarily promotes bilirubin photoisomerization rather than nanoparticle-mediated oxidation. In addition, adsorption of Mn³⁺ ions on the surface of T-Fe₃O₄ allowed modulation of the bilirubin degradation rate: while degradation was accelerated in the absence of light, UV irradiation significantly reduced catalytic efficiency due to electron transfer from the excited state of the nanoparticles to the Mn ions, thereby inhibiting their oxidation. Although this tunable behavior is mechanistically relevant, dependence on UV irradiation and the absence of biological validation limit direct therapeutic translation.

### In vivo evaluation of light-source performance in bilirubin phototherapy

Table 2 summarizes the experimental conditions and bilirubin degradation outcomes reported in the selected in vivo studies, enabling direct comparison of light parameters and biological models.

**Table 2:** Experimental conditions and degradation outcomes reported for in vivo bilirubin photodegradation studies

Entry	Bilirubin concentration (µM)	Wavelength (nm)	Duration time (h)	Animal model	Degradation (%)	Ref
1	342	465–470	5	Gunn rats	21–39	[35]
2	171	400–500	8	Wistar rats	78	[48]

**Fig. 8:**
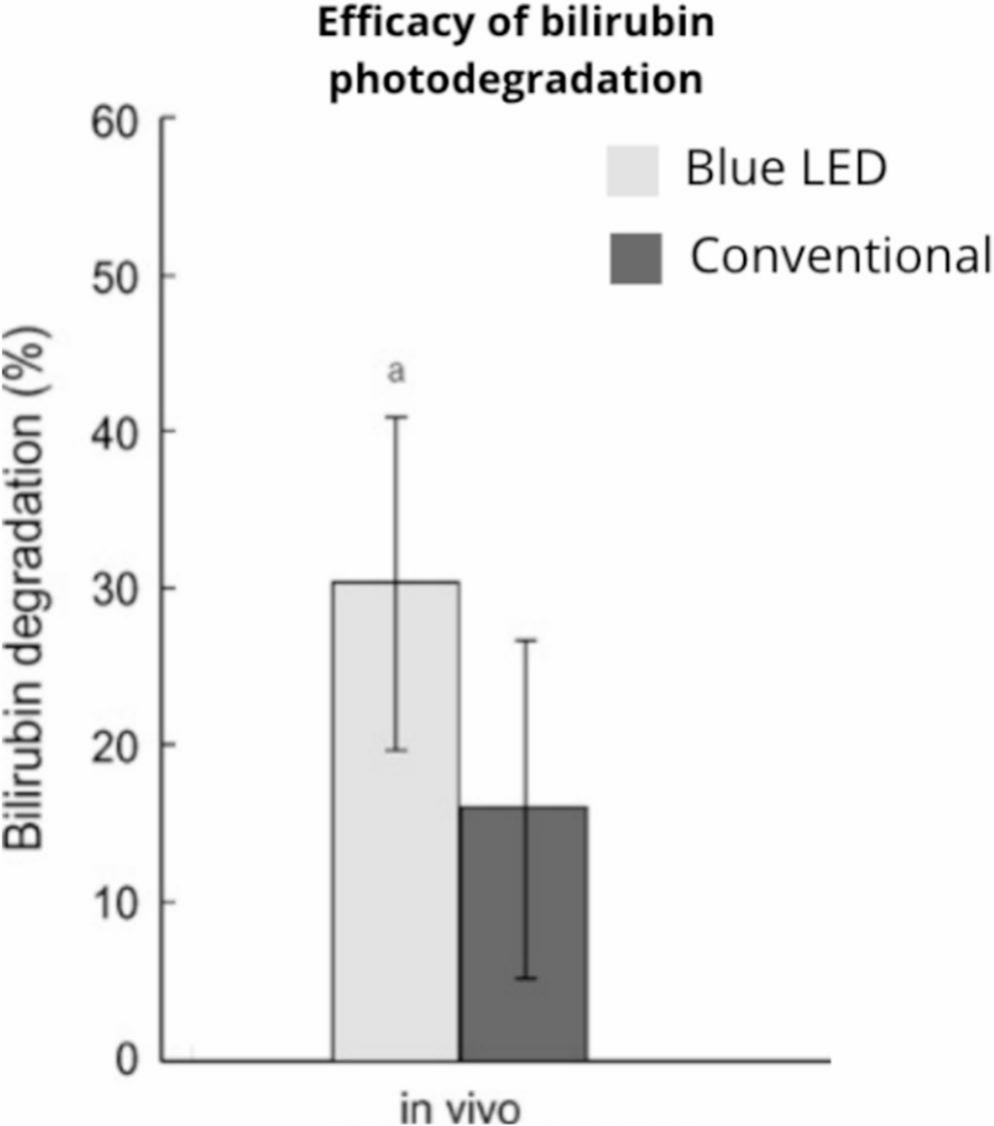
In vivo bilirubin reduction achieved with a high-intensity blue LED system compared with a conventional quartz–halogen device. Bars represent mean values ± SD. **ᵃ** *p* < 0.05 versus the conventional system. Adapted from [35].

Chang et al. [35]. investigated the efficacy of a high-intensity blue LED phototherapy system using an in vivo model of hyperbilirubinemia (Fig. 8; Table 2, entry 1). The experiments were performed in jaundiced Gunn rats exposed to both the LED system and a conventional quartz–halogen device under identical conditions of distance and exposure time. The LED-based system demonstrated superior efficacy in reducing bilirubin levels. Because the devices differed in spectral distribution and irradiance, the improved response cannot be attributed exclusively to the LED source itself. These findings highlight that optimized irradiance within the bilirubin absorption spectrum is a critical determinant of phototherapy effectiveness, supporting the potential of high-intensity blue LED systems as a more efficient alternative for the treatment of neonatal hyperbilirubinemia. Nevertheless, the Gunn rat model does not fully reproduce neonatal skin optics, bilirubin distribution, or clinical treatment conditions, and confirmation in neonates remains necessary.

In 2009, Sebbe and colleagues [48] developed and characterized a novel LED-based phototherapy device designed to enhance bilirubin photodegradation compared with conventional fluorescent lamp sources (Table 2, entry 2). The authors tested the prototype in an animal hyperbilirubinemia model, demonstrating that the LED device delivered higher irradiance within the blue spectrum and promoted more efficient bilirubin breakdown than the traditional lamps. Consistent with previous LED studies, these results indicate that therapeutic performance depend primarily on the spectral overlap with bilirubin absorption and the irradiance delivered to the target, rather than on the light-source technology itself. The system’s improved performance was evidenced by a greater reduction in bilirubin levels under LED illumination, suggesting that light wavelength and intensity are critical for optimizing photodegradation outcomes. According to the authors, these findings indicate that high-intensity LED phototherapy devices may offer a more effective approach for treating neonatal jaundice by accelerating bilirubin photodegradation compared with older light sources.

## Conclusions and future directions

This review highlights that bilirubin phototransformation is a complex, wavelength-dependent process governed by both photoisomerization and photooxidation mechanisms, with significant implications for the optimization of neonatal phototherapy. Current evidence supports photoisomerization as the primary therapeutic pathway, whereas the relative contribution and clinical relevance of photooxidation depend on irradiation conditions, oxygen availability, and the experimental medium. LED-based systems operating within the blue–green spectral region generally show improved performance relative to conventional light sources; however, this advantage is primarily associated with greater spectral overlap, irradiance control, and reduced heat generation rather than with LED technology itself. Moreover, the optimal wavelength may vary according to photon flux, bilirubin binding, tissue optical properties, and the biological matrix. Optoacoustic, fluorescence-based, and miniaturized photometric systems offer promising strategies for real-time or point-of-care bilirubin monitoring. Nevertheless, most remain at the proof-of-concept stage and require validation in neonatal samples, comparison with reference clinical methods, and evaluation across different skin characteristics, gestational ages, and bilirubin concentrations.

Nanotechnology-based approaches, including semiconductor nanoparticles and hybrid nanocomposites, have emerged as promising tools to enhance bilirubin photodegradation through catalytic and energy-transfer mechanisms. These approaches differ fundamentally from conventional phototherapy because they frequently promote oxidative degradation and may require UV irradiation, direct contact with nanomaterials, or controlled adsorption. Their reported efficiencies should therefore not be directly compared with blue-light phototherapy without considering differences in mechanism and experimental conditions. Although bilirubin absorbs strongly in the blue region near 460 nm, longer blue–green wavelengths may penetrate tissue more effectively and promote greater lumirubin formation under normalized photon flux. Therefore, the wavelength producing the highest in vitro photochemical efficiency may not necessarily provide the greatest clinical response. Clinical efficacy depends on the balance between bilirubin absorption, tissue penetration, irradiance delivered to the skin, and the formation of rapidly excretable photoisomers.

Clinical translation will depend on demonstrating biocompatibility, catalyst stability, the toxicological safety of degradation products, scalability, and effectiveness in complex biological matrices. Future research should evaluate multi-wavelength phototherapy systems capable of dynamically combining blue and blue–green irradiation to balance bilirubin absorption, tissue penetration, and lumirubin formation. Integration with real-time optical monitoring could enable continuous assessment of bilirubin response during treatment. In this context, AI-assisted models may integrate bilirubin concentration, optical signals, irradiance, exposure time, gestational age, and skin-related variables to predict treatment response and optimize irradiation parameters. These developments may support feedback-controlled phototherapy systems that automatically adjust wavelength, irradiance, and treatment duration according to the measured bilirubin dynamics. However, such closed-loop platforms will require robust clinical datasets, transparent and validated algorithms, safety constraints, and prospective neonatal evaluation before clinical implementation. Future investigations should also bridge the gap between in vitro optimization and clinical application, with particular emphasis on safety, scalability, reproducibility, and integration into existing neonatal care systems. Overall, the convergence of photophysics, nanotechnology, and biomedical engineering, real-time sensing, and data-driven treatment control is expected to drive the next generation of phototherapy strategies, enabling more efficient, safer, and personalized management of neonatal hyperbilirubinemia.

## Data Availability

No datasets were generated or analysed during the current study.
